# Catalase in Testes and Epididymidis of Wistar Rats Fed Zinc Deficient Diet

**DOI:** 10.4103/0250-474X.51959

**Published:** 2009

**Authors:** S. Bedwal, S. Prasad, N. Nair, M. R. Saini, R. S. Bedwal

**Affiliations:** Cell Biology Laboratory, Department of Zoology, University of Rajasthan, Jaipur-302 004, India; 1 Radiation Biology Laboratory, Department of Zoology, University of Rajasthan, Jaipur-302 004, India

**Keywords:** Catalase, testes, epididymis, and zinc deficiency

## Abstract

Catalase activities have been evaluated in testes and caput and cauda epididymis of Wistar rats fed on zinc deficient diet for 2 and 4 weeks. The enzyme activity has been measured as chromic acetate formed by heating of dichromate (in acetic acid) in presence of H_2_ O_2_ with perchromic acid as an unstable intermediate. Observed non-significant increase in catalase activity in testes as well as in caput and cauda epididymis of 2 weeks experiments has been related to low levels of H_2_ O_2_ produced in two organs whereas significant (P<0.01/0.001) increase in catalase activity in 4-weeks experiments indicate for increased oxidative stress due to phagocytotic activity of Sertoli cells in testes and damaged spermatozoa in epididymis. Thus, zinc deficiency increases catalase activity in testes and epididymis.

Zinc is indispensable element for growth, reproduction, development, differentiation, immune and antioxidant functions, gene expression, DNA synthesis, hormone synthesis, storage and release of neurotransmitters, memory, visual processes and apoptosis[1,2]. Cardinal symptoms of zinc deficiency are retarded growth and hypogonadism[2]. Oxidative stress has been suggested to be an early effect of zinc deficiency rather than a simple reflection of zinc deficiency-induced tissue pathology[3]. Reactive oxygen species (ROS) or free oxygen radical (FOR) are normally generated by Sertoli cells that cause alteration in cellular structures and induces morphological changes in spermatids during spermiogenesis[4] and controlled amount of ROS is essential for capacitation and acrosome reaction[5]. In fact, analysis of superoxide radical generated by rat epididymal spermatozoa has revealed a two component process involving leakage from sperm mitochondria at complex I and II and a plasma membrane NAD(P)H oxidoreductase whose activity is regulated by zinc[6,7]. In spite of these pivotal roles played by ROS in reproduction they have been, in general, implicated in injuries of testes and spermatozoa leading to infertility. Catalase (CAT) has been reported from testis, seminal plasma, and spermatozoa and in peroxisomes of rodent Leydig cells[8]. A sharp eight-fold drop in CAT activity in rabbit testes from day 64 to 101 of age (this period corresponds to completion of Leydig cell maturation and on set of pre-pubertal spermatogenic cycle including spermatogenesis) suggests for a possible relationship between testosterone and catalase activity[9]. Low levels of CAT mRNA, evaluated by northern blot and *in situ* hybridization, were detected in testis (primarily in peritubular and intersitial cells) and epithelial cells of epididymis of normal rats whereas on efferent duct ligation, in spite of thinning of germinal epithelium, an increase in testicular CAT mRNA has been reported[10]. The present study was designed to study the effects of zinc deficiency on catalase of testes and caput and cauda epididymidis.

Colony bred Wistar rats of 30d of age were used in the present study and were fed synthetic diet with either 100-ppm zinc or <1.0-ppm zinc for two and four weeks. The synthetic diet was prepared according to Wallace *et al*[11]. Thirty animals were divided into three groups: ZC (control; 100 ppm zinc in the diet), ZD (zinc deficient; <1.0ppm zinc the diet) and PF (pair fed; 100 ppm zinc control diet but the amount of the diet was equal to the amount consumed by zinc deficient group). All the animals were provided with demineralized water *ad libitum*.

Animals were autopsied after two and four weeks under light ether anesthesia. Testes and epididymidis (caput and cauda) were excised, cleaned off of extraneous tissues, weighed on electronic balance, and processed for catalase estimation by the method of Sinha[12]. The absorbance was measured on Carl Zeiss Spekol ZV (Germany) spectrophotometer. Statistical significance of data was evaluated by Student's t-test. Level of significance selected was 0.01. The experiments were approved by the Departmental Ethics Committee.

Catalase activity increased in testes and in caput and cauda epididymis of zinc deficient (ZD) rats as compared to their respective control (ZC) and pair fed (PF) group animals from 2 and 4-w experiments (figs. 1–3). However, the level of significance varied from tissue to tissue and interval of experiment. The increase was significant (P<0.01) in testes of ZD rats as compared to respective PF and ZC whereas the increase in testes of 2ZD rats as compared to 2PF group was non-significant. Further, the increases in enzyme activities in caput and cauda epididymis were also significant (P<0.01) in zinc deficient (ZD) group as compared to respective PF and ZC groups except in caput epididymis from two weeks experiments where the increase was non-significant (figs. 1–3).

**Fig. 1:**
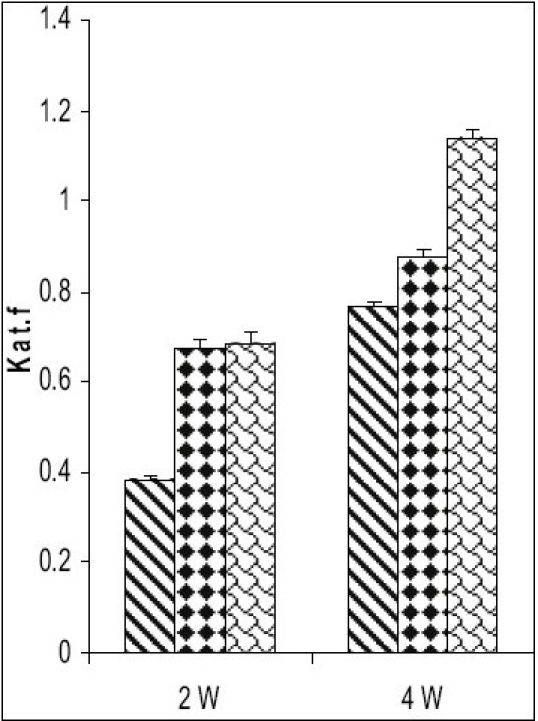
Catalase activity in testes of Wistar rats fed on zinc-deficient diet ZC is control 
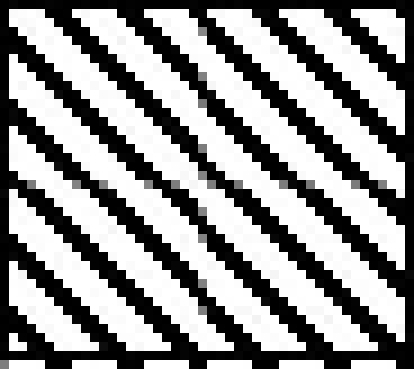
, PF is pair fed 
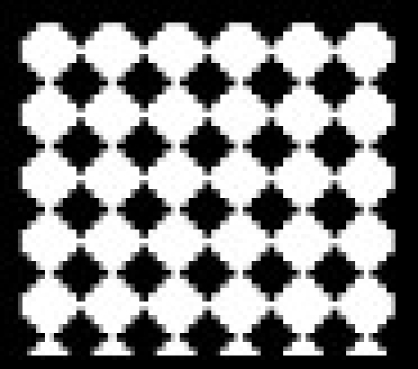
, ZD is zinc-deficient 
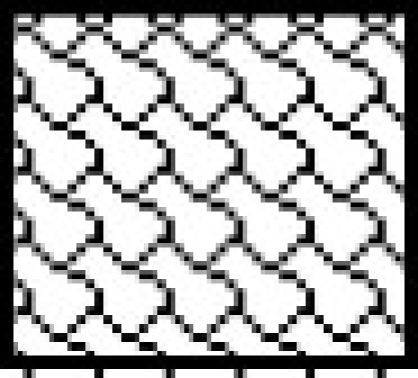

**Fig. 2:**
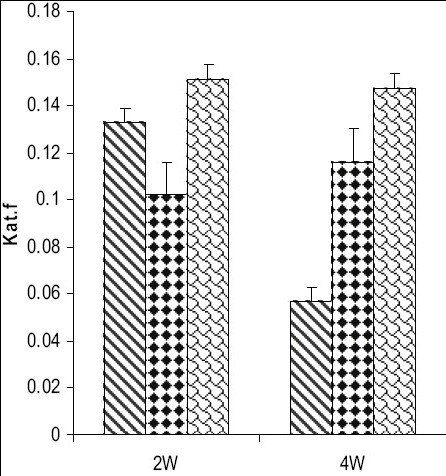
Catalase activity in caput epididymis of Wistar rats fed on zinc deficient diet. ZC is control 
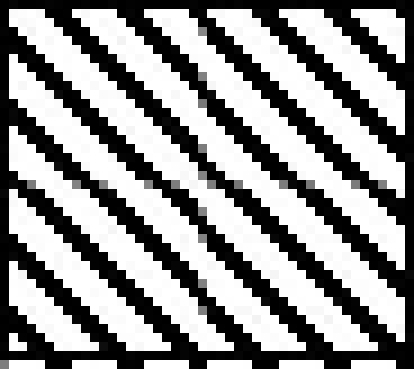
, PF is pair fed 
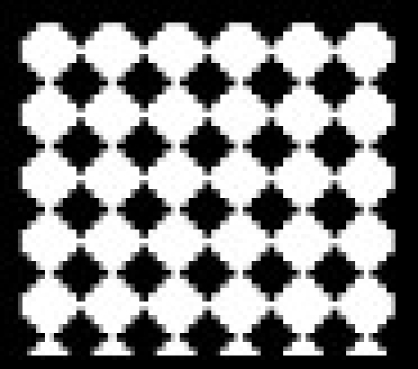
, ZD is zinc-deficient 
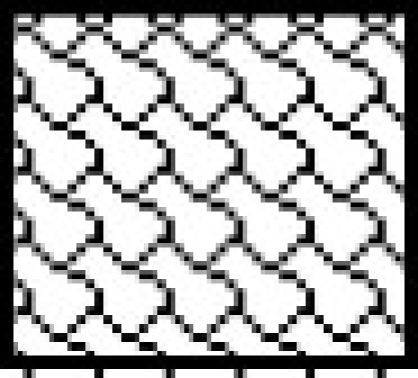

**Fig. 3:**
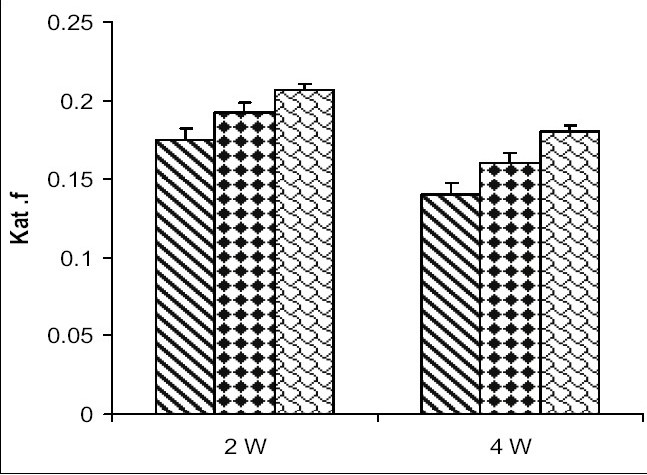
Catalase activity in cauda epididymis of Wistar rats fed on zinc deficient diet. ZC is control 
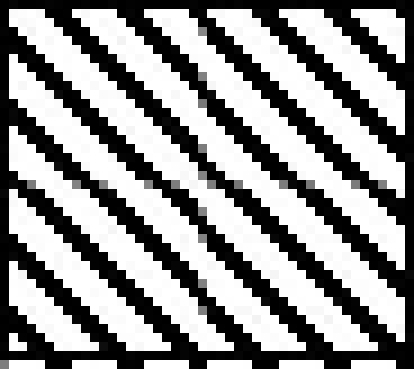
, PF is pair fed 
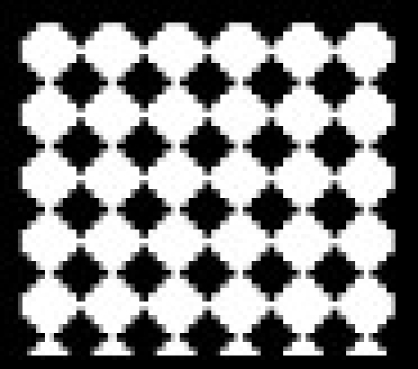
, ZD is zinc-deficient 
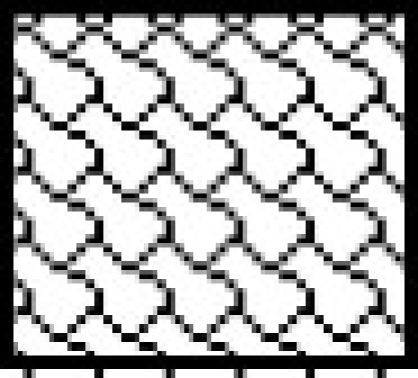

Zinc has close relationship with endocrine system being essential for testosterone synthesis and spermatogenesis. Its deficiency causes atrophy of seminiferous tubules, failure of spermatogenesis[13] and decreased testosterone secretion in rat. Hypogonadism in zinc deficient rats is not because of hypothalamus-pituitary dysfunction but is because of irresponsiveness of Leydig cells to gonadotrophin[14,15]. In fact, steroid hormone receptors have zinc finger motifs that act as DNA binding domain of transcription factors[16]. Thus zinc deficiency impairs the functions of steroid receptors and decreases sex steroid action[16]. Short-term (7 d) zinc deficiency is reported not to cause overt signs of oxidative damages to cell components of testis of rats[17] while long-term deficiency leads to increased oxidative stress[18]. CAT activity increased in testes and in caput and cauda epididymidis of zinc deficient (ZD) rats as compared to their respective control (ZC) and pair fed (PF) group animals from 2 and 4-w experiments. However, the level of significance varied from tissue to tissue and interval of experiment. The non-significant increases in CAT activities from testes and caput and cauda epididymis after 2 w indicates no overt signs of oxidative damage as reported earlier by Oteiza *et al.*[17] and histological observations of this laboratory[13]. Phagocytosis is always associated with respiratory burst in which high level of O_2_^·-^ are produced[19,20]. This feature is particularly important in Sertoli cells because they phagocytose germ cells debris and considerable amount of late spermatid residual cytoplasm during spermatogenesis and a large number of degenerated germ cells after injury to testes[21]. CAT mRNA activities have been shown to be primarily present in peritubular and interstitial cells of testes throughout its development and are efficiently translated[10]. CAT bearing peroxisomes have been described in Leydig cells of rodents[8] but CAT activity is absent in primary spermatocytes and round spermatids[22]. CAT activity is increased in interstitial cells as compared to other cells of seminiferous tubules by testosterone administration[23] and zinc treatment[24]. This suggests for a possible relationship between testosterone and CAT and zinc and CAT. In the present study, zinc deficiency induced testosterone depletion should have decreased CAT activity in testes of zinc deficient rats. On the contrary, an increase has been observed. The increased activity may be for removal of increased hydroperoxides[25] by increased phagocytosis of Sertoli cells. Studies have also indicated that zinc deficiency causes lipid peroxidation[26] to generate free radicals and endogenous peroxides which are highly reactive and causes changes in cell membrane permeability and possibly necrosis[27]. Authors[26,28] have reported changes in antioxidant enzymes (total SOD, Cu-Zn SOD, MnSOD, GSH, GPx and γGT) after zinc deficiency indicating an increased sensitivity to oxidative stress as a consequence of increased ROS generation and/or decreased zinc dependent antioxidant processes.

Several studies indicate that ROS are involved in damaging sperm membrane and defective sperm functions[29]. Although epididymis is characterized by a little blood supply and a low epididymal temperature (both reduce oxidative stress by limiting pO_2_ and cell metabolism), yet the epididymal fluid contains high level of toxic H_2_ O_2_[30]. Thus, inactivation of H_2_ O_2,_ while spermatozoa transit through or are stored in epididymis appears to be essential for prevention of premature capacitation. Simultaneously, it has to protect its own epithelium from ROS. Hence the organ expresses and secretes a variety of antioxidant enzymes including CAT[31]. The accumulation of superoxide anion is eliminated by SOD (specially MnSOD) which generates H_2_ O_2_. Studies have revealed changes in SOD activity and other scavenging enzymes[26,28] in epididymidis after zinc deficiency Hence, an increased CAT activity has been observed in caput and cauda epididymis of zinc deficient rats. Thus, CAT seems to be an important antioxidant enzyme in testis as well as in caput and cauda epididymis of zinc deficient rats.
